# Exploring the connection between childhood trauma, dissociation, and borderline personality disorder in forensic psychiatry: a comprehensive case study

**DOI:** 10.3389/fpsyg.2024.1332914

**Published:** 2024-02-23

**Authors:** Claudia Scognamiglio, Antonia Sorge, Giovanni Borrelli, Raffaella Perrella, Emanuela Saita

**Affiliations:** ^1^Department of Human Sciences, Guglielmo Marconi University, Rome, Italy; ^2^Department of Psychology, Università Cattolica del Sacro Cuore, Milan, Italy; ^3^Department of Psychology, University of Campania Luigi Vanvitelli, Caserta, Italy

**Keywords:** childhood trauma, dissociation, borderline personality disorder, forensic psychiatry, risk assessment, rehabilitation

## Abstract

This case study examines the complex relationship between childhood trauma, dissociation, and Borderline Personality Disorder (BPD) within the context of forensic psychiatry. It focuses on a young murder defendant named “Paul,” who has experienced various traumatic events, including childhood maltreatment and domestic violence. These experiences have led to dissociative states marked by high emotional intensity, particularly of an aggressive nature, and impaired impulse control, resulting in violent behavior during dissociative episodes. The study employs advanced assessment tools like Raven’s Standard Progressive Matrices (SPM), the Millon Clinical Multiaxial Inventory-III (MCMI-III), and the Level of Service/Case Management Inventory (LS/CMI) to gain a comprehensive understanding of Paul’s psychopathological condition, risk factors, and rehabilitation needs. The LS/CMI assessment highlights a high risk of recidivism, mainly influenced by family relationships, educational challenges, interpersonal connections, and aggressive tendencies. To address the multifaceted needs of individuals like Paul, the study emphasizes the importance of using transdiagnostic models for trauma and dissociation. This approach informs tailored treatment programs that include processing past traumatic experiences, improving self-identity, nurturing healthy relational patterns, and enhancing emotional regulation. Although this study is based on a single case, it serves as a model for integrating assessment tools and theoretical-clinical models in the field of forensic psychiatry. Understanding the intricate dynamics of childhood trauma, dissociation, and BPD is crucial for making informed decisions, conducting risk assessments, and developing rehabilitation programs within the justice system. Future research should expand the scope of cases and further validate assessment tools to advance our understanding of this complex relationship.

## Introduction

Data collected from research on inmates found an interesting link between trauma and criminal behavior (Welfare and Hollin, 2015; Peltonen et al., 2020) highlighting how childhood trauma could be part of the trajectory toward the later use of violence. Childhood trauma, including sexual, physical, and emotional abuse, as well as neglect, is prevalent among individuals with Borderline Personality Disorder (BPD) (Dadomo et al., 2022; Tate et al., 2022; Leichsenring et al., 2023); thus, it is also considered a potential environmental causative factor in the development of BPD (Ball and Links, 2009). The enduring consequences of early adversities give rise to specific internal object relations, which shape the individual’s psyche. These internal object relations, as explained by Nemeroff (2012), align closely with the Karpman’s Drama Triangle (Karpman, 1968), in which individuals can unconsciously switch between three distinct roles (i.e., the victim, the abuser, and the rescuer), influencing the individual’s mental state and behavioral patterns. Understanding the connection between childhood adversity and BPD involves considering affective lability and alexithymia as key factors (Edwards et al., 2021). Individuals who have greater difficulties identifying their emotions are more likely to exhibit signs of a personality disorder, with specific reference to BPD (Berenbaum, 1996). Recent research conducted by Pourmohammad et al. (2021) found that individuals with BPD had significant difficulty recognizing and expressing their feelings compared to a healthy control group. This “blind spot” in emotional awareness is particularly pronounced in individuals who have endured childhood psychological trauma. Within the Development-Based Trauma Framework, psychological trauma is categorized into multiple types (Kira et al., 2013; Kira, 2022). While type I trauma (e.g., a traffic accident) can cause the development of PTSD, type II trauma (e.g., repeated sexual abuse over time) and type III (i.e., ongoing complex trauma) can cause the development of complex PTSD (cPTSD). cPTSD in the ICD-11 is a broader diagnosis that includes the core PTSD symptoms plus an additional set of “disturbances in self-organization” (DSO) symptoms (Hyland et al., 2018). Exposure to prolonged and repeated multiple traumatic experiences over the course of childhood might also have deleterious effects on children in terms of their potential for violent conduct (Zettler, 2021; Craig et al., 2023).

In the realm of forensic psychiatry, the assessment of criminal responsibility, the risk of recidivism, and social dangerousness takes center stage. The criteria for the insanity defense may vary, yet there is a consensus that an impaired mental state can substantially affect an individual’s accountability for criminal acts and their potential to pose a threat to society (Meynen, 2013; Mandarelli et al., 2019; Hartvigsson, 2023). A recent meta-analysis, focusing on defendants’ characteristics, found that a clinical judgment of not criminally responsible is predominantly associated with a psychiatric history and the presence of a psychotic disorder (Kois and Chauhan, 2018). Conversely, individuals deemed socially dangerous are more frequently affected by disorders within the schizophrenia spectrum or personality disorders such as BPD and Antisocial Personality Disorder (ASPD). Understanding the implications of these personality disorders on criminal responsibility and social dangerousness is of paramount importance.

The symptomatology of BPD can be frequently exacerbated by the presence of dissociative symptoms. According to Al-Shamali et al. (2022), a lack of emphasis on dissociation in BPD assessment may lead to incorrect diagnoses or an underestimation of significant comorbidities. Dissociative symptoms include memory loss (amnesia) for significant events or time periods events, and people; experiences of depersonalization or derealization; misperception of people and things as distorted and unreal; blurred sense of identity; and hearing voices (which stem from various dissociative parts of the personality) (Mosquera and Steele, 2017; Al-Shamali et al., 2022). Dissociation, as a complex mental process arising in response to traumatic experiences or extreme stress, disrupts the typical integration of various aspects of consciousness (American Psychiatric Association, 2022). It is well known that experiencing multiple traumatic events during childhood can prompts children to develop dissociation as a self-regulatory mechanism, significantly affecting their mental and behavioral functioning (Sharma et al., 2021). The profound impact of dissociation on individuals’ mental states and their potential for violent or criminal behavior should not be underestimated.

Researches have provided evidence for the connection between early traumatic experiences, antisocial behavior, aggression, and dissociation (Spitzer et al., 2001, 2003; Ruiz et al., 2008; Zavattini et al., 2017). Individuals diagnosed with Dissociative Identity Disorder (DID) may experience distinct personality states (i.e., ‘alters’) some of which may exhibit dangerous or criminal behaviors. Dissociative amnesia (DA), a common feature of DID, can lead individuals to forget their actions in crimes, raising significant questions about their criminal responsibility.

Moreover, childhood trauma, along with other historical and situational factors, can interact with brain development (Masson et al., 2015; Cross et al., 2017). The immaturity, dysfunction, or damage of certain brain areas, such as the prefrontal cortex, can substantially affect an individual’s ability to control emotions, think ahead, and learn from their actions. This understanding is particularly relevant in the context of juvenile offenders, emphasizing the far-reaching consequences of brain development on behavior (Lewis et al., 2004).

The assessment of criminal responsibility, social dangerousness, and the risk of recidivism necessitating ongoing examination (Gkotsi and Gasser, 2016). With individual freedom at stake, it is imperative to enhance the precision of our judgments, given the profound implications for public safety and the rehabilitation of individuals in the justice system.

This study aimed to assess a young murder defendant with a history of multiple traumas, focusing on his criminal responsibility, recidivism risk, and social dangerousness. We considered various factors, both static and dynamic, to provide psychiatric insights into his past and future criminal behavior.

## Methods

### Participant and procedure

In this study, we present the case report of a defendant for the murder of a man unknown to him. The case (referred to using the pseudonym Paul) was an 18-years old boy without previous official criminal record. When questioned about the crime, he states that he does not remember anything that happened.

The assessment procedure took place in a private room of the prison. The evaluation was provided by a forensic psychologist with more than 20 years of experience in the field of legal reporting. It consisted of four session of interviews and psychological testing allowed by the judge. We ensured the internal validity through the use of standardized assessment tools (Perrella and Russo, 2018). Additionally, we established a discussion group consisting of four experienced professionals in the field of correctional psychology, providing valuable insights and perspectives and then enhancing the overall validity of the assessment process.

### Case report

#### Family history

Paul’s family history is characterized by numerous traumatic events. His parents separated shortly after his birth. He never had any relationship with his father, who has been in prison for a violent offense since Paul was a child. His mother was an alcoholic and has psychiatric health problems. She manifested disconcerting and ambivalent behaviors throughout his entire childhood, conceivably contributing to the development of a disorganized attachment type. Presently, she is under the care of a public health facility and is receiving anti-psychotic therapy. Because of his father’s imprisonment and the mother’s mental health issues, Paul was raised by his grandparents before being turned over to social services and put in a juvenile residential facility. During adolescence, he returned to the family home with his mother. The woman was aggressive, often escalating to physical violence toward him. His mother’s romantic relationships with men were dysfunctional and involved domestic violence.

#### Education

Paul was held back twice due to absences and low grades. He discontinued his studies during his third year of high school due to his incarceration. As regard to his relationships with peers, Paul reports a complete absence of a social support network.

#### Clinical history

Since early childhood, Paul showed frequent episodes of aggressive behavior toward peers, his mother, and her partners. His aggressive conduct has determined four compulsory health treatments during adolescence. Paul arbitrarily terminated the prescribed psychiatric therapy.

#### Incarceration

While in prison, Paul engaged in self-harming behavior for instrumental purposes. He has formed positive relationships with the correctional staff. However, within a short span, two instances of aggression occurred toward fellow inmates, one of whom seemingly without any apparent reason.

### Measures

*Raven’s Standard Progressive Matrices* (SPM; Raven, 1936; Raven et al., 2000) is a standard measure for intelligence. The SPM consists of 60 items divided into 5 sets of 12 items each. Each item requires completing a series of figures by identifying the missing element in relation to the presented model, following a criterion of increasing difficulty. The final score reflects an individual’s age-related intellectual abilities, irrespective of their level of education. The outcome provides insights into the strategic utilization of logical processes in an attempt to achieve secondary gains (i.e., malingering).

*The Millon Clinical Multiaxial Inventory-III* (MCMI-III; Millon, 2006) is psychological assessment tool designed to evaluate and diagnose various psychological and psychiatric disorders in adults. The MCMI-III consists of 24 clinical scales: 14 personality disorder scales and 10 clinical syndrome scales. It provides information on several axes, including personality styles, clinical syndromes, and severe personality pathology, making it valuable in clinical and forensic settings for diagnosing mental health conditions and guiding treatment decisions.

*The Level of Service/Case Management Inventory* (LS/CMI, Andrews et al., 2004) is a semi-structured interview about the offender’s risk factors, criminogenic needs, and degree of responsiveness, but it is also a comprehensive case management tool (Bonta and Wormith, 2013). LS/CMI consists of 11 sections. The first section allows the identification of the subject’s level of reoffending through the assessment of eight sub-components (criminal history, education/employment, family/marriage, leisure time, companions, alcohol/drug problems, pro-criminal attitude and orientation, antisocial personality pattern). Despite the Italian validation study of the tool is still in progress, the Italian translation of LS/CMI was approved by the authors and publisher Multi-Health Systems Inc. (MHS), then was used to evaluate the subject of the present research.

## Results

### Mental state evaluation

Throughout the forensic evaluation, Paul displayed a high degree of cooperation. Paul’s emotional state’s expression was moderate and effectively managed during the interviews. His emotional state fluctuated depending on the topic of conversation. He exhibited noticeable tremors in his hands and face, particularly intensifying during discussions related to his family and the offense. He reported experiencing anxiety-related tachycardia. In contrast, he was calm and relaxed when discussing his positive relationship with a correctional staff member. No perceptual disturbances were observed. Paul’s thought process appeared logical and coherent, with no disturbances in thought form. His mnemonic abilities remained intact in terms of orientation in time and space. Despite this, he is unable to describe a specific episode of his mother’s aggressive behaviors; moreover, he has no recollection of the offense or of the violent conduct in prison. In summary, Paul’s psychodynamics appear to be characterized by dissociative mental states triggered by emotional hyperactivation, often of an aggressive nature. This condition seems to impair significantly his impulse control.

### Raven’s standard progressive matrices

Paul achieved an SPM score of 66, which places his IQ within the 1st percentile with a 95% confidence interval. In other words, there is a 95% probability that his IQ falls between the extreme lower level and the below-average level compared to individuals in his age group (See Table 1).

**Table 1:** Raven’s progressive matrices.

	A series	B series	C series	D series	E series	Total
% of participant	100	83	58	50	25	63
% of reference age group	98	96	84	86	60	84

Raw Score	IQ	Percentage	IC 90%	IC 95%
38	66	1	63–79	61–80

### Millon clinical multiaxial inventory – III

**Figure 1:**
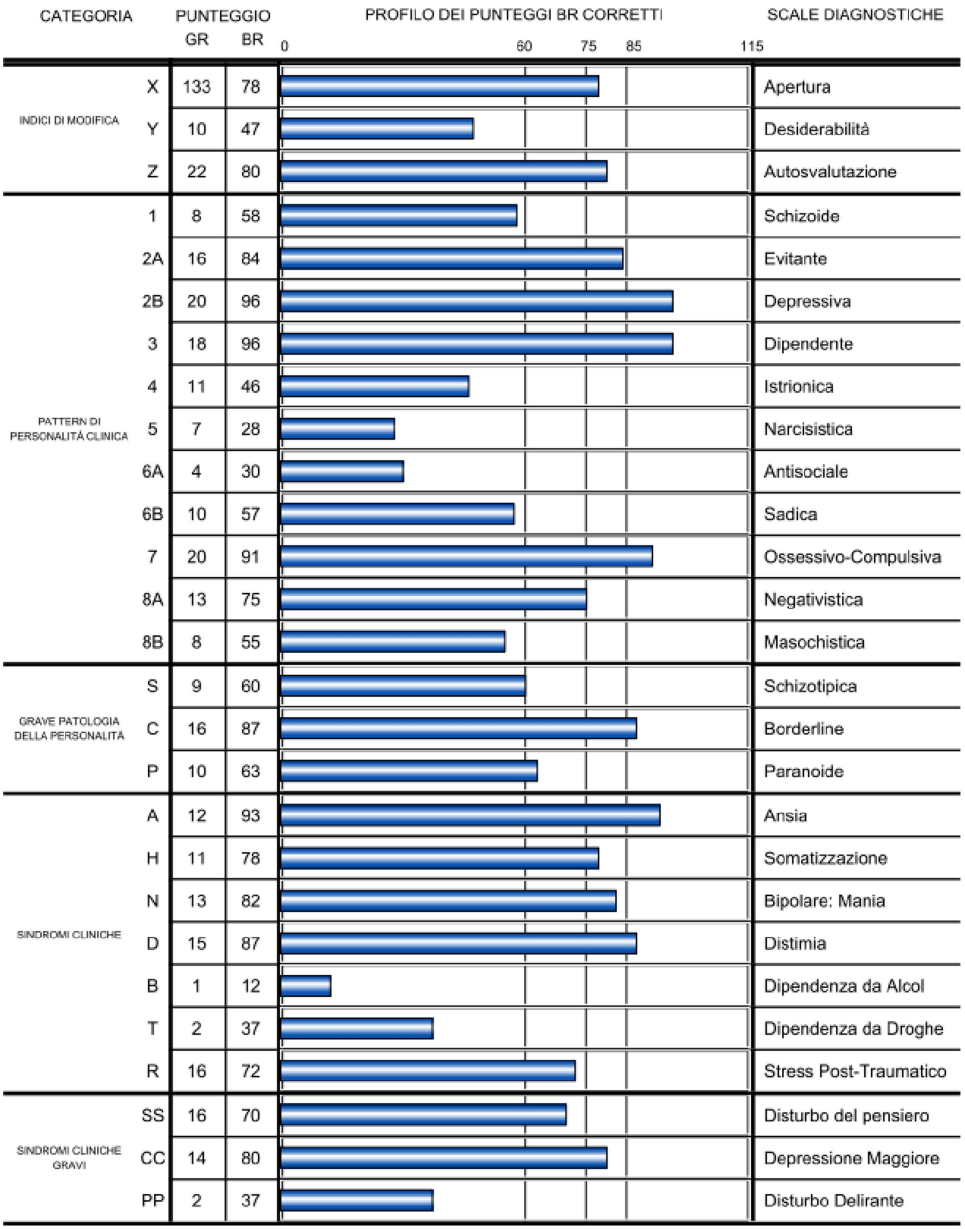
MCMI-III profile.

Paul’s MCMI-III profile (See Figure 1) revealed several noteworthy findings. There was a clinically significant elevation (i.e., BR ≥ 75), on Scale X (= 78) and Scale Z (= 80). This elevation suggests a propensity for scoring higher on MCMI-III scales compared to the patient’s clinical status.

In terms of personality pattern scales, Paul exhibited clinically significant elevations on the following: Scales 2A (Avoidant; BR = 84); and, 8A (Negativistic - Passive-Aggressive; BR = 75).

Turning to the Clinical Syndrome Scale, Paul scored significantly high on Scale H (Somatoform; BR = 78), and Scale N (Bipolar-maniac; BR = 82). In the category of Severe Clinical Syndrome, his scores were clinically significant on Scale CC (Major Depression; BR = 80).

Furthermore, pervasive scores (i.e., BR ≥ 85), were observed on the following clinical personality patterns: Scale 2B (Depressive; BR = 96); Scale 3 (Dependent; BR = 96); and Scale 7 (Compulsive; BR = 91). Additionally, Paul displayed a pervasive score in the category of severe personality pathology, particularly on Scale C (Borderline; BR = 87).

Regarding the Clinical Syndrome Scale, Paul exhibited pervasive scores on Scale A (Anxiety; BR = 93), and Scale D (Dysthymia; BR = 87).

#### Level of service/case management inventory – Section 1

##### Criminal history (CH)

Paul has no previous criminal records. While incarcerated, he carried out two aggression toward fellow inmates. CH subcomponent scores 1: very low risk (See Table 2).

**Table 2:** Risk/need profile of level of service/case management inventory (LS/CMI; Andrews et al., 2004).

Risk/Need	CH	EE	FM	LR	CO	ADP	PA	AP	Tot
Very high	8	8–9	4	-	4	7–8	4	4	30+
High	6–7	6–7	3	2	3	5–6	3	3	20–29
Medium	4–5	4–5	2	1	2	3–4	2	2	11–19
Low	2–3	2–3	1	-	1	1–2	1	1	5–10
Very low	0–1	0–1	0	0	0	0	0	0	4–0

##### Education/Employment (EE)

Paul has no employment history and discontinued his education in his third year of high school due to low academic performance and non-attendance. His education was permanently interrupted due to his incarceration. His interactions with schoolmates were marked by isolation, bullying, and discrimination. EE subcomponent scores 7: high risk.

##### Family/Marital (FM)

His father has a criminal record for a violent crime. His mother suffering from chronic psychosis. Paul was exposed to domestic violence due to his mother partners. Maternal grandparents are no longer living. The relationship with his sister is described as “cold”. FM subcomponents scores 4: high risk.

##### Leisure/Recreation (LR)

Although Paul maintains a clean-living space and engages in recreational activities such as strategy games, music, drawing, and watching “anime” TV series he does not participate in organized activities within the prison, suggesting he could make better utilization of his time. LR subcomponent scores 2: high risk.

##### Companions (CO)

While in prison, Paul is in contact with individuals involved in criminal activities. He claims to have no friendships. He reports familiarity with fellow inmates but does not consider them friends. CO subcomponent scores 3: high risk.

##### Alcohol/drug problem (ADP)

Paul’s mother was an alcoholist. He recalls consuming alcohol in her presence, but he asserts that he never developed an addiction and never used drugs. ADP subcomponent scores 2: low risk.

##### Procriminal Attitude/Orientation (PA)

Paul expresses feelings of guilt for his actions but claims no recollection of the moment of the attack on the victim. He acknowledges the importance of societal conventions and the need to work but did not consistently attend school, engage in work, or participate in social group activities. PA subcomponent scores 1: low risk.

##### Antisocial pattern (AP)

Paul was diagnosed with BPD, characterized by poor self-control and violent behaviors during emotional dysregulation. His family background includes parental mental health problems and involvement in the criminal justice system. He experienced adjustment difficulties during adolescence and faced numerous compulsory health treatments due to family quarrels. He has records of aggressions. AP component scores 3: high risk.

**Figure 2:**
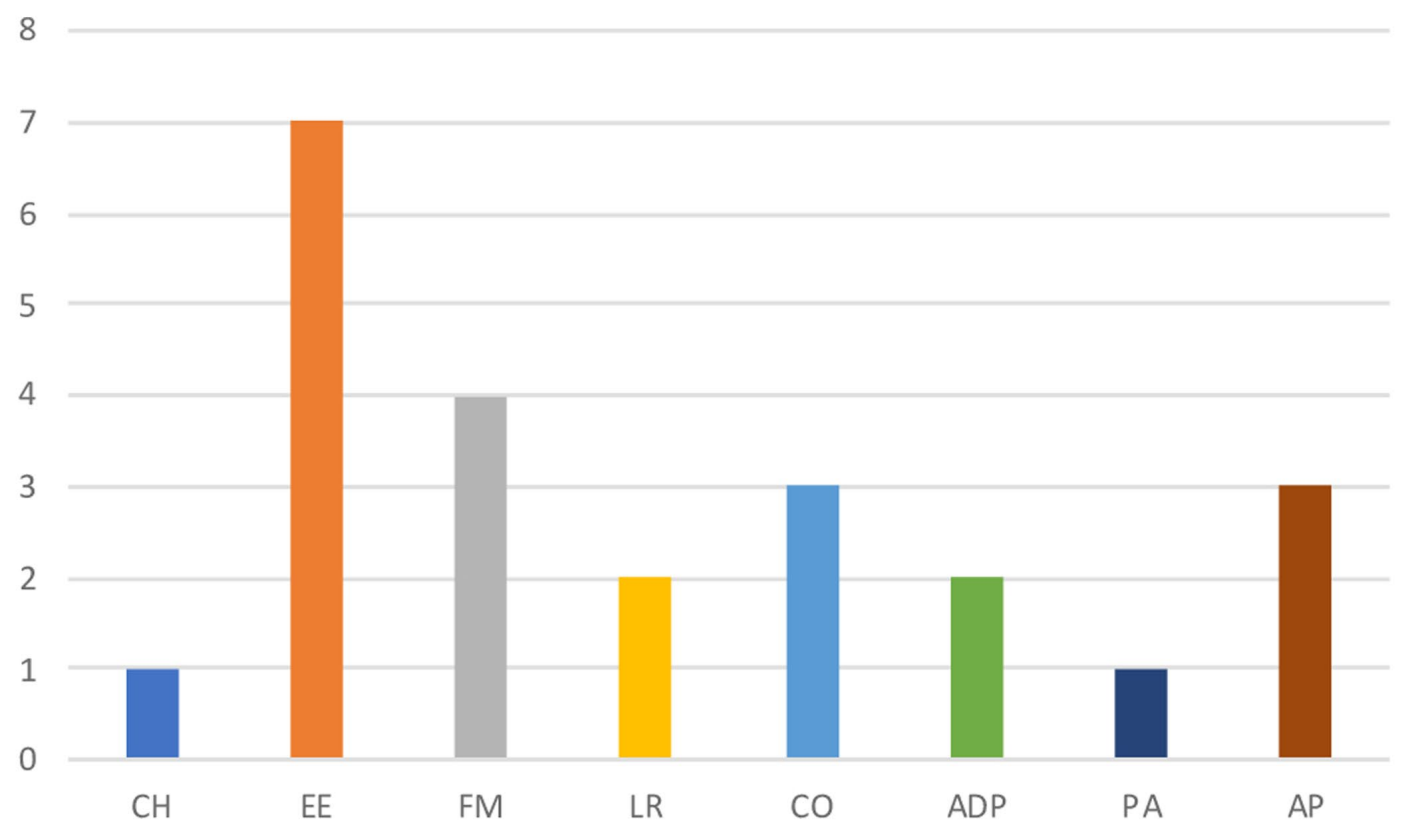
Histogram with the eight subcomponents of LS\CMI.

The overall risk assessment conducted using the LS/CMI yielded a high risk of recidivism (LS/CMI = 23). The subcomponents associated with the highest risk levels are education/employment (EE), family/marital (FM), and companions (CO), as illustrated in Figure 2.

## Discussion

Through forensic assessment, Paul received a diagnosis of BPD and DA. People with BPD may experience loss of reality when confronted with stressors (Mosquera and Steele, 2017). Paul’s psychopathological manifestations are rooted in his complex life history, marked by childhood maltreatment, relational traumas, and domestic violence.

As a result of the evaluation, Paul was assessed as not criminally responsible. Legal precedents, such as Supreme Court Judgments No. 9163/2005 and No. 188/2020,^1^ have established that personality disorders can result in incapacity when there is a demonstrable link between the mental disorder and the offense (Fornari, 2006; Perrella and Russo, 2018).

The risk assessment was carried out using the LS/CMI model, adhering to the principles of Risk-Need-Responsivity (RNR; Andrews et al., 1990, 2011; Wormith and Bonta, 2020; Sorge et al., 2022). It showed that the main risk factors for reoffending are Paul’s family relationships (FM), school and work-related challenges (EE), the absence of a friendship network (CO), and his heightened aggression with limited frustration tolerance (AP). These factors indicated a high risk of recidivism, particularly given the occurrence of heterodirected aggressive acts during dissociative states. On the positive side, he displayed an absence of a criminal history (CH), and no substance abuse (ADP). Moreover, Paul reported a sense of belonging attributed to his positive relationship with certain prison officers. It highlights the significant role that connections with prison staff can play in the process of rehabilitation (Sorge et al., 2021).

Given the complex psychopathological framework, it is crucial to employ transdiagnostic models of trauma and dissociative symptoms to comprehensively comprehend the clinical dynamics and develop tailored treatment plans. For instance, the DBTF model by Kira et al. (2013) and Kira (2022) underscores the significance of identifying the type of trauma to inform precise interventions.

Concerning dissociative symptoms, the 4-Dimension model (4-D; Frewen and Lanius, 2014; Lanius, 2015) categorizes traumatic stress symptoms into Time, Thought, Body, and Emotion dimensions, encompassing both normal and trauma-related altered states of consciousness (TRASC). TRASC is relevant for understanding trauma-related disorders and factors contributing to the development and persistence of trauma-related symptoms (Bækkelund et al., 2018).

By employing these models, it becomes feasible to conceptualize Paul’s psychopathological condition, establish a precise diagnosis, and formulate appropriate re-educational and treatment recommendations. It is worth noting that while the primary diagnosis is BPD, there exists substantial clinical variability in the disorder, further complicated by the comorbidity with DA. Given the risk factors identified through the LS/CMI assessment (EE, FM, AP, CO), particularly in the context of dissociation, it is essential to work on improving Paul’s interpersonal relationships within the family, school, and friendships. Enhancing study skills, and imparting self-control and anger management techniques is crucial. Addressing Paul’s attachment-related adverse experiences, which severely impact mentalization, brain development, and stress response systems, is equally paramount. These traumas, experienced repetitively over time, constitute complex trauma, exacerbating his post-traumatic symptomatology, which is expressed through TRASC manifestations. To design an effective individualized rehabilitation intervention, interpreting results from valid instruments through these theoretical-clinical models is essential. This approach restores individual subjectivity and aligns with the RNR model. To enhance interpersonal relationships, clinical intervention should focus on processing past traumatic experiences, strengthening self-identity, fostering healthy relational patterns, and improving the ability to recognize one’s mental and emotional states, regulate emotions, and build resilience in the face of stress. Developing insight into one’s mental and physical states can help Paul implement functional coping strategies during moments of heightened stress. The union of an individualized re-educational intervention with clinical work is pivotal to managing the risk of reoffending, particularly concerning the regulation of behavior and emotions during dissociative states. Paul should also assess his improved emotional management in school and work contexts, and in the context of his attachment figure relationships. In summary, the data gathered through the assessment procedure offered valuable insights into Paul’s clinical and psychosocial condition, facilitating a precise risk assessment and the formulation of a rehabilitation program.

### Study limitations

The current study has several significant limitations that need to be considered. Firstly, it focused on a single case of BPD and DA, which cannot comprehensively represent the whole clinical population. While individual cases provide valuable insights into clinical practice, it is important to acknowledge methodological constraints. Nevertheless, we presented an assessment procedure that can be replicated.

It is worth noting that, despite numerous studies demonstrating the effectiveness and reliability of the LS/CMI, the validation process is still ongoing in Italy. However, the tool is already accessible in most other countries. Future research should aim to address this limitation by presenting data from a larger sample, and reinforcing the connection between Trauma, BPD, and criminal responsibility. Proposing qualitative investigations could be valuable, considering the uniqueness of the clinical condition (Saita et al., 2022).

## Conclusion

The evaluation of criminal responsibility and the potential for social dangerousness in cases involving BPD holds significant importance within the realm of forensic psychiatry. The link between BPD and childhood trauma often results in dissociative symptoms that disrupt the integration of consciousness states. This, in turn, may exposes individuals to aggressive or criminal behaviors triggered by stressors that reactivate trauma-related memories.

The complexity of psychodynamics showcased in the present case study underscores the necessity of integrating assessment and case management tools with theoretical-clinical models informed by the latest scientific literature. This integration aims to establish a foundational protocol for psychodynamic assessment procedures in the field of forensic psychiatry, as currently, there are no well-defined and legally established practices.

### Future directions

Acknowledging the study’s limitations, several recommendations for future research are delineated. Firstly, integrating additional case studies that explore variations in trauma experiences, symptomatology, and risk factors is proposed to augment the generalizability of findings. Secondly, the ongoing validation process of the LS/CMI in Italy highlights the need to spread the use of assessment tools more widely. Finally, the suggestion to undertake longitudinal studies for insights into long-term trajectories, along with collaboration with legal and ethical experts to establish precise guidelines, is advanced. These proposed directions collectively aim to refine interventions, elevate evidence-based practices, and establish comprehensive protocols within the field of forensic psychiatry.

## Data availability statement

The data analyzed in this study is subject to the following licenses/restrictions: the authors cannot share the raw data that underpins our conclusions because it is bound by privacy restrictions related to the protection of the subject. Requests to access these datasets should be directed to raffaella.perrella@unicampania.it.

## Ethics statement

Written informed consent was not obtained from the individual(s) for the publication of any potentially identifiable images or data included in this article. This is because, in adherence to ethical guidelines and in accordance with legal requirements, the assessment in this study was conducted with the explicit permission of the presiding judge overseeing the case under evaluation. This judicial authorization ensured that the assessment was carried out in a lawful and ethical manner, respecting the rights and due process of the individuals involved. No written statement of informed consent was obtained. To protect their privacy, any sensitive data is omitted. In this way, their identity cannot be traced.

## Author contributions

CS: Formal analysis, Methodology, Writing – original draft, Writing – review & editing. AS: Formal analysis, Methodology, Writing – original draft, Writing – review & editing. GB: Formal analysis, Methodology, Writing – original draft, Writing – review & editing. RP: Conceptualization, Data curation, Formal analysis, Methodology, Supervision, Writing – review & editing. ES: Conceptualization, Formal analysis, Methodology, Supervision, Writing – review & editing.

## Footnotes

^1^Available in: https://www.brocardi.it/notizie-giuridiche/disturbo-della-personalita-integra-vizio-mente-solo-incide-sulla/2356.html. Accessed 3^rd^ November 2023.
